# Perspectives on Smoking Initiation and Maintenance: A Qualitative Exploration among Singapore Youth

**DOI:** 10.3390/ijerph120808956

**Published:** 2015-07-31

**Authors:** Mythily Subramaniam, Shazana Shahwan, Restria Fauziana, Pratika Satghare, Louisa Picco, Janhavi Ajit Vaingankar, Siow Ann Chong

**Affiliations:** Research Division, Institute of Mental Health, Buangkok Green Medical Park, 10 Buangkok View, 539747, Singapore; E-Mails: Shazana_MOHAMED_SHAHWAN@imh.com.sg (S.S.); Restria_Fauziana@imh.com.sg (R.F.); Pratika_Satghare@imh.com.sg (P.S.); Louisa_Picco@imh.com.sg (L.P.); Janhavi_Vaingankar@imh.com.sg (J.A.V.); Siow_Ann_Chong@imh.com.sg (S.A.C.)

**Keywords:** smoking, peers, family, health-risks, policy, Asian

## Abstract

Studies among adolescents have shown that several important interpersonal, intrapersonal and environmental factors are associated with smoking behaviour. The current qualitative research project aimed to explore the determinants of smoking initiation and maintenance, from a youth perspective, among young people who smoked, living in a multi-ethnic Asian country. Focus group discussions (FGDs) were conducted with youths in Singapore in youth-friendly and accessible locations. Young people, from a variety of social contexts—varying on age, gender, ethnicity and educational level, were included in the study. All FGDs were conducted in English and participants were recruited using a mix of network and purposive sampling. All FGDs were audio recorded and transcribed verbatim. The data were analyzed using qualitative content analysis, allowing themes to emerge from the data with the goal of answering the research question. Ninety-one youth smokers (54 males, 37 females), aged between 14 to 29 years, participated in the study. The majority were males (59%) and of Chinese ethnicity (52%). Participants identified multiple personal, social, and familial influences on young adults’ smoking behaviors. Peer and family influences, as well as risk minimization, played a key role in smoking initiation and maintenance. While young people were aware of policies that restricted smoking, these did not directly affect their decision to start smoking. The theory of triadic influence provided a promising theoretical framework to understand smoking initiation and maintenance in a sample of young adult smokers from a multi-ethnic Asian country. It also provides actionable information for initiatives to prevent smoking in young people, which includes their perspectives and emphasizes an inclusive approach without stigmatizing those who smoke.

## 1. Introduction

The higher prevalence of smoking among youth and young adults is an almost universal finding in epidemiological studies that examine smoking [1,2]. Tobacco use often begins and becomes established during adolescence [3]. In the USA, about 4000 youths under 18 years of age experiment with smoking everyday and more than 1500 youths become daily smokers [4]. Data further suggest that the average age of smoking initiation in North America is 12.1 years [5,6]. Once smoking behavior is entrenched, cessation is difficult and studies have found that the likelihood of quitting was higher in those who initiated smoking at an older age [7]. Even low consumption and short exposure to smoking can lead to adolescent smokers experiencing symptoms of nicotine dependence [8]. Consequently, preventing adolescent uptake of cigarette smoking remains an important public health issue [9,10] and a key component of efforts to reduce the overall prevalence of smoking and smoking-related morbidity and mortality.

Singapore is an island city-nation off the southern tip of the Malay Peninsula. In 2013, the population of Singapore was just under 5.4 million, of which 3.85 million were Singapore residents. Of these residents, 74.2% were of Chinese descent, 13.3% were Malays, and 9.2% were of Indian descent while 3.3 % of the resident population comprised other ethnic groups [11]. Prohibition of smoking in certain places in Singapore was first introduced in 1970 and has been progressively extended to virtually all indoor places and areas where the public congregate. This act (Smoking Prohibition in Certain Places), enforced by the National Environment Agency, renders it an offence to smoke in prohibited places listed under the act, and smoking under the age of 18 years is also an offence in Singapore. Singapore also meets the requirements of the World Health Organization (WHO) Framework Convention on Tobacco Control (FCTC), which includes a ban on tobacco advertising, promotion and sponsorship [12].

Surveys among the adult population have shown a slight increase in smoking rates in Singapore over the past 10 years, despite various policy and legislative related measures being put in place that aim to reduce smoking. Results from the 2010 National Health Surveillance Survey found that the prevalence of daily smoking among adult Singapore residents (aged 18 to 69 years) was 14.3% [13] and had increased slightly since previous surveys in 2007 and 2004 [14,15], when it was 13.6% and 12.6%, respectively. An epidemiological survey conducted in Singapore in 2010 among adults aged 18 years and above found the prevalence of current smoking to be 16% and it was associated with a younger age, the male gender, Malay ethnicity, and lower educational status [16].

Studies among adolescents have shown that several important intrapersonal (age, ethnicity, risk perception, *etc*.), interpersonal (parental and peer smoking, parental attitudes, *etc*.), and environmental factors [17,18,19] are associated with tobacco use and particularly with smoking behaviour. Findings consistently point towards the role of peers and family on youth smoking behavior [20,21], while evidence is emerging that environmental determinants, such as smoke free laws [22] also play a role. While considerable research has been undertaken in Western countries on these issues, few studies have explored this in the Asian context. Local studies have largely focused on establishing the prevalence of smoking and risk factors of smoking behaviour, and none to date have tried to understand why certain young people initiate smoking and continue to smoke despite the adverse health outcomes. Thus, the aim of this qualitative research project was to explore the context of smoking initiation and maintenance, from youth perspective, among young people living in a multi-ethnic Asian country.

## 2. Methods

### 2.1. Sample

Focus group discussions (FGDs) were conducted with youths in Singapore who smoked; where smokers were defined as those who had smoked at least once in the past month [23]. Young people from a variety of social contexts—varying on age, gender, ethnicity, and educational level, were targeted for inclusion. All FGDs were conducted in English and participants were recruited using a mix of network and purposive sampling. Ninety-one youth smokers (54 males, 37 females) aged between 15 to 29 years participated in the study. In all, 12 mixed group (in terms of gender and ethnicity) FGDs were conducted from September 2013 to June 2014, and each FGD lasted on average 90 minutes. All the FGDs were conducted in a youth friendly location which was easily accessible. The study was approved by the relevant ethics committee (National Healthcare Group Domain Specific Review Board, Singapore) and all participants provided written informed consent.

### 2.2. Data Collection

All FGDs were conducted by two team members; a facilitator and a note-taker. The responsibilities of the facilitator and the note-taker were to: (a) explain consent procedures and ensure that consent forms were signed prior to beginning the FGD, and (b) obtain demographic information. In addition to conducting the discussion, the facilitator ensured that inconsistent, vague or cryptic comments were probed for understanding, while the note-taker took careful notes of the group process, discussion and seating arrangements. The facilitators (Mythily Subramaniam, Shazana Shahwan, Restria Fauziana) were trained and experienced in qualitative research methodologies. FGDs were conducted using a common FGD guide to ensure standardization across the focus groups. The following themes were explored: (1) How and why did they start smoking including attitudes and experiences towards smoking before initiation; (2) Other people’s response to their smoking habits and reasons for continuing to smoke; and (3) Awareness and attitudes towards smoking prevention and cessation programs. Participants were allowed to raise additional issues which they considered important towards the end of the FGD. Data collection ended when no new perspectives were revealed and thereby data saturation was reached. Demographic and smoking related data was collected using a structured questionnaire to provide a more complete understanding of the sample characteristics.

### 2.3. Data Analysis

All FGDs were audio recorded and transcribed verbatim and the facilitator checked the transcripts for consistency. The data was analyzed using qualitative content analysis thus allowing themes to emerge from the data with the goal of answering the research question [24]. The study used “conventional content analysis” as described by Hsieh and Shannon [24], wherein data were read word by word and those that identified key concepts were highlighted to derive codes. Codes were sorted into categories and emergent categories were used to organize and group codes into meaningful clusters.

Four authors (Mythily Subramaniam, Shazana Shahwan, Pratika Satghare and Restria Fauziana) each independently conducted an analysis of a sub-set of transcribed FGDs. The data was broken down into smaller units and assigned labels or codes based on the content they represented. Following this, the coded data was grouped together according to concepts to form themes [25]. Code terms were discussed and refined and after a second level of analysis of the same sub-set of data, a codebook was constructed. Consensus was reached through discussion and iterative review of codes and themes. A fifth author was available to consult if a consensus could not be reached about the categories; however, this was not needed. Three authors (Mythily Subramaniam, Shazana Shahwan and Pratika Satghare) coded the same transcripts (three transcripts in all) using the codebook developed and inter-rater reliability was compared; Cohen’s kappa coefficient was established to be 0.74. The three authors then coded the transcripts independently. NVivo 10 was used for data analysis.

## 3. Results

The age of the participants ranged from 15–29 years (Median—24 years), and the majority were males (59%) and of Chinese ethnicity (52%). The duration of smoking varied between 1 to 16 years. Socio-demographic and smoking related correlates of the participants are shown in Table 1. Participants identified multiple personal, social and familial influences on young adults’ smoking behaviors and these are described below.

### 3.1. Smoking to Fit In: Role of Peers

Peer socialization is a process whereby adolescents take on the values and behaviors of the “group” in order to be accepted [26]. Peer socialization can be overt, as in peer pressure, or perceived, where the adolescent accepts or changes attitudes and behavior based on perceived group norms that may or may not be real. There were differing perceptions on the phenomenon of ‘peer pressure’ to smoke. While some vehemently denied that there was any form of external pressure on them to smoke and were categorical that it was their own decision to initiate smoking, others did report feeling pressurized. Pressure was exerted in the form of repeated offers of cigarettes, ridiculing their refusal to smoke, questioning their reasons for not wanting to smoke, *etc.* As one of them said,

*“This was back when I first started university. They were like, ‘Oh you should try this, don’t be such a… prude, try lah try lah won’t die one.’ Then ok ok try and then I felt like $#% (expletive).” (The language used by participants iscolloquial Singaporean English or Singlish)(‘lah’ in Singlish is a discourse particle, it is used commonly in conversations to indicate the speaker's attitude, or to structure their interactions with other participants)*.

**Table 1:** Socio-demographic characteristics of focus group discussion participants.

Variable	Sub-Groups	Range	Median
Age (in years)		15–29	24
Duration of smoking (in years)		1–16	5
		**N**	
Sex	Female	37	
	Male	54	
Ethnic group	Chinese	47	
	Malay	29	
	Indian	14	
	Others	1	
Education Level	Completed Primary Education	2	
	Secondary Education (Ongoing)	5	
	Completed Secondary Education	23	
	Vocational Institute	10	
	Completed Pre University or		
	Junior College	6	
	Diploma	28	
	University Degree	15	
	Others	2	

A more consistent theme that emerged was that of indirect pressure to conform to social norms. This was expressed as watching people in their clique smoke and desiring to join them, or wanting to be a part of a group where many members were smokers, thus, taking up smoking to blend in, feeling embarrassed that they were the only non-smokers in a group, although there was no overt pressure from the group. As one young person said,

*“How I picked it up. Okay, my sister, she had to babysit me. So in order for her to get out of the house, I’ll have to tag along. So naturally, I would have to mix around with her common clique so that’s where…”*.

### 3.2. Smoking as a Social Tool

Smoking was perceived by most young people as a largely social activity. Though some stated that they also enjoyed smoking by themselves, most participants indicated that they tended to smoke more in the company of friends. Smoking was often described as a means to develop broader social networks and even as a means for networking. Smokers perceived other smokers as being “friendlier” and more approachable, as highlighted below.

*“I do feel that smokers are generally friendlier and you actually make more friends during smoke breaks. Especially in university you don’t know anyone, you just start smoking and then you’re like, ‘Oh, didn’t know you’re a smoker. Next time we’ll smoke together.’ You make a lot of friends along the way, even when you’re in a party, you’re invited to all those club and all.”* 

Young people also felt that their friendships with other smokers were more meaningful and deeper and that they could discuss and share more about their life and problems with them than with their non-smoking friends. Participants also highlighted the role of alcohol, usually in the context of social gatherings, in facilitating smoking.

### 3.3. Smoking as a Coping Tool

Young people highlighted other perceived benefits of smoking, including that it serves as an aid for alleviating stress, tiredness and fatigue, helps them refocus their thoughts and concentrate better during study or work related sessions. “Smoke-breaks” were also seen as a way to get a break without being perceived as shirking from work.

*“It relaxes my mind. I need more of cigarettes, I realize, when I’m angry, when I’m frustrated, annoyed at something. That’s when I take more of cigarettes. Then somehow I’ll be more calm.”* 

### 3.4. Flavored Cigarettes, Curiosity and Other Reasons for Initiating Smoking

Young people also stated that flavored cigarettes were one of the reasons they were tempted to smoke. The sweet/minty taste of some of the flavored cigarettes or the cool sensation experienced when smoking these cigarettes was reported as enjoyable. Some also felt that flavored cigarettes were “less strong”, “less harsh” and thus “safer” to smoke, which led to their initiating smoking with these cigarettes.

A few young people said that curiosity about the act of smoking and the experience associated with it led them to experiment with smoking. Some young people felt that they started smoking as an act of rebellion against parents or schools and as a protest against the discipline imposed on them or the demand for academic excellence. Just one participant perceived smoking as a skill, and the only reason for taking it up was to blow a perfect ring. As the young person expressed it,

*I just tried it because I wanted to make ‘O’s with it.”* 

### 3.5. Family Influences and Practices

While most young people stated that their family members had little influence on their decision to smoke, a number of them endorsed the fact that they were exposed to smoking or cigarettes prior to smoking due to the smoking habits of immediate or extended family members. While more young people endorsed that they were comfortable with the act or smell associated with smoking and generally did not have a negative opinion of smokers, some said that they disliked the smell of smoking and were personally against smoking before they started smoking. Some even expressed surprise at their own volition to start smoking in spite of their initial opinion, and were unable to enunciate the reason for the change. A few young people were categorical in stating that the smoking habits of their family did influence their smoking. In the words of two young people,

*“How I was brought up and stuff like that was one of the key to me smoking and stuff like that.” “Because I got introduced to, as in my parents and their friends they all smoke so when I was younger I was exposed to such stuff so eventually I think I got okay with it.”* 

Some young people stated that their first cigarette was offered by an older sibling and very few stated that the first cigarette was offered by a parent. Many felt that smoking was a way of bonding with their sibling, while others talked about how they shared cigarettes with siblings or helped each other by buying cigarettes or colluded to keep their parents from becoming aware of their smoking habits.

Another theme that emerged strongly was that young people felt that parents who themselves smoked had no right to advise them or tell them to not to start or stop smoking. As a young person said, somewhat angrily,

*“I won’t take $#% (expletive) from my dad because he smokes so he can’t say that I cannot and… if he ever finds out lah. And also, I dunno, like my sister used to smoke so she also cannot say anything.”* 

Most young people talked about rules regarding smoking in their homes only after their parents found out about their smoking. While some felt that their parents turned a blind eye as long as they did not smoke at home, others spoke about how their parents had set clear rules that they would not allow smoking in the house. Very few smokers mentioned that their parents either shared cigarettes with them or even purchased cigarettes for them. As one person said about his father,

*“Sometimes he will offer me cigarettes as well like, my relatives have some leftovers that they buy from overseas and just he pass it to me I’m like – ‘Okay, thanks.’”* 

### 3.6. Perceived Health Risks of Smoking

Personal health concerns were generally not introduced spontaneously during the discussions. While participants were aware of the health risks associated with smoking and listed them down frequently in a free listing exercise as one of the ‘not so good things’ associated with smoking, this was not mentioned as a specific concern to them spontaneously. When asked directly about their reaction to the information available widely on the adverse health outcomes associated with smoking, most participants agreed that they were aware of them. However, they simultaneously minimized that risk. Participants generally avoided using names of specific diseases as perceived health risks and focused more on symptoms of poor health caused by smoking. Loss of stamina, cough and breathlessness were often highlighted by the participants.

*“Yeah kind of, because I also like… I told you I dance and I exercise on the side and I notice like my health really sucks now…I can’t run as fast!”* 

In terms of risk minimization, our findings were very similar to that reported by Oakes *et al.* [27], and, thus, we use their terms for the first three similar categories identified: “skeptic” beliefs, “worth it” beliefs, and “jungle” beliefs. The last category was similar to that identified by Helweg-Larsen *et al.* [28] of “risk-delay”. Participants felt that risks associated with smoking were exaggerated and were not convinced that there was enough local research to support these findings (skeptic-beliefs). As one participant said,

*“Because they say that smoking give you lung cancer. My late grandfather smokes but he died of natural causes, no cancer, no nothing.”* 

Many felt that all behaviors were associated with some risk and life is dangerous in many ways (jungle beliefs) and they were willing to take the risk associated with smoking as it balanced the ‘benefits’ afforded to them (worth-it beliefs).

*“But once you get out on the road you get banged by car, anytime. The rate of people dying, on the road is more than the people who actually smokes.”* 

*“It’s like drinking those carbonated drinks as well. I mean it does give you if you drink too much you get diabetes for example yeah so anything in excess you get illnesses. So for me it’s like a choice that I’m willing to risk, yeah. Something that I want to do.”* 

Many young participants exhibited ‘risk delay’ beliefs as they felt that the adverse outcomes associated with smoking were likely to occur only at a later time, *i.e.*, when one is older not when they were young. They also felt that they would have quit before the adverse outcomes occur. As one young person expressed it,

*“Around our age group we don’t really see our friends passing on like probably we’ll just regret when we are older and stuff.”* 

### 3.7. Cost and Other Adverse Effects of Smoking

Participants seemed much more concerned with the financial burden engendered by smoking—as the cost of cigarettes was considered high for those not working or working part-time. Another risk that emerged spontaneously was that of ‘cosmetic risks’- the toll smoking took on their appearance which varied from girls worrying about wrinkles to those of both gender reporting stained fingers and teeth, as well as the unpleasant mouth and body odor associated with smoking. Participants reported spending considerable amount of money to get rid of teeth staining as well as use of mints and perfumes to mask mouth and body odors.

Stigmatization and stereotyping by others was seen as one of the risks of smoking. Young people felt that they were ‘labeled’ as smokers and people had pre-conceived notions about smokers that were not favorable. People in certain jobs, like teachers or those representing an organization in front-line operations, felt that their customers would judge them and/or their organization adversely if they found out they were smokers. Others felt that people “stared” at them, “held their noses”, “waved smoke away” with their hands or tried to distance their children even though they were smoking at a good distance from them. They also felt that media usually depicted “bad guys” as smokers which further perpetuated the belief that smokers were bad. As one young person expressed it:

*“It’s like there is this guy named Tom, then some people they will go like, ‘Oh this is a smoker named, Tom’ not, ‘this is a guy named Tom he also happens to be a smoker’ it goes a different way, so it’s like they judge you because you are smoking.”* 

### 3.8. Harmful Effects of Smoking to Others and the Environment

Most smokers understood the risk caused by second hand smoke to non-smokers and acknowledged that it affected the health of those around them. They also talked about not smoking inside the home as the space is enclosed, trying to blow the smoke in the opposite direction or momentarily holding in the smoke in their mouths while others especially small children pass by and walking away from them. They felt that while smoking was a choice that they had made willingly and had weighed the harm, it was not fair to expose those who were not smoking to the same harm. As one person said,

*“Yeah, and it’s bad for people around me especially kids. I cannot take it when I smoke and then there are kids around me. That, aiyah I feel bad.”(‘Aiyah’ is an expression commonly used in Singapore to mean ‘sigh’).* 

Some also talked about the negative effects on the environment as a whole and issues pertaining to air pollution. Girls expressed concerns about the harm that smoking could cause to their babies and in the event of pregnancy. Most girls were categorical that if they got pregnant they would quit smoking. Young men were similarly determined to limit their smoking at home or cut down on smoking if their partners were to get pregnant.

### 3.9. Attitudes towards Smoking Policies

All the smokers were well aware of the various policy related steps that had been taken to prevent or reduce smoking. This included knowledge of pricing of cigarettes, sale of full packs and not loose cigarettes, creation of smoke free zones, extension of non-smoking areas, smoking only in designated areas, fines for smoking in non-designated areas, *etc.* Young people had divided opinions about the policies. While some felt that it made them responsible smokers, others felt that some measures were ‘too much’. Many young people felt that the increase in the price of cigarettes did not affect their smoking habits as they just cut down on other essentials or took up part-time jobs. Young people felt that the policies were focused on deterring smoking but did not take the person who smoked into consideration. They suggested that such measures should go hand-in-hand with emphasis on programs on smoking cessation. Some of them also wondered why the policy did not encompass a complete ban on smoking while others talked about the difficulties and disadvantages associated with such prohibitions. Statements like the following reflect these sentiments:

*“Yeah, yeah, there’s one zone maybe a few estates where you’re totally not allowed to smoke, not even a yellow box.”* 

*“I think that they are trying to control the supply side of it but they never look at the demand side of it because I think the demand side of it is a more pressing issue like maybe they could like create some more programs like for smokers to learn how to minimize or reduce then followed by quitting the habit.”* 

## 4. Discussion

The study identified that for young people who smoked, the immediate social environment, *i.e.*, family and peer networks, as well as the risk/benefit ratio as assessed by them played a central role in smoking initiation and maintenance. Smoking related policies also came up in the discussion but was seen as a distant influence on smoking behavior. These major themes indicated the applicability of the theory of triadic influence (TTI) to explain the findings of the study.

The TTI [29] is an ecological model of health behavior, which states that all behaviors are influenced by an interaction of genetic and environmental factors. This model has been used widely to explain substance related behaviors [30,31,32]. The TTI focuses on three streams of influence; intrapersonal (genetic, gender, ethnicity, age), social (peer and parental influences) and cultural-environmental (community characteristics, legislation and policy) that influence intentions and behaviors. These influences in turn are divided into ultimate, distal, and proximal level causes. Ultimate-level causes are broad and relatively stable, and individuals have little control over them. Proximal-level predictors while influenced by the distal and ultimate factors are under the control of an individual [29].

In our study, the social and the cultural-environmental streams emerged as strong predictors of smoking behavior. Social ultimate level was represented by parenting styles and the perception of family members as role models. Smoking related practices within families, including role modelling smoking, and facilitating access to tobacco were influential in smoking uptake among youth. These findings are consistent with a number of other studies that have found that parent and sibling smoking is a strong and significant predictor of risk of smoking by children and young people [33]. Family, especially siblings, both as a direct source of cigarettes, as well as initiators of smoking, has been previously reported in other studies [30]. Social proximal themes were that of social normative beliefs. The study found evidence that peer influences have a significant role in smoking initiation. Similar to other recent studies, we found that the peer influence was not one of overt pressure to smoke but one that was influenced by peer selection and peer socialization [30].

In terms of the cultural-environmental stream, ultimate level factors like policy were mentioned and young people were cognizant of the restrictions imposed by them. While these influenced their smoking practices in terms of physical space, a strong influence was not perceived in terms of initiation. On the other hand, distal and proximal factors which included young people’s expectations and evaluations of the cost and benefit of tobacco consumption dominated the discussion. The distinct risk minimization observed in this study is in line with other research that shows that individual appraisals of risk are different from that of experts [34], and that smokers use a number of strategies to minimize or deny those risks [27,28]. Interestingly young people who smoke expressed their concern for the risks caused to others and the environment as a result of their smoking. These findings are somewhat different from that of a qualitative study of Danish and American smokers who minimized the risk to others [28] and emphasized cultural differences associated with smoking behavior. These perceived risks were however outweighed by the perceived benefits of smoking, such as stress relief, improved concentration, reduced fatigue, and opportunities to socialize.

Another theme that emerged from the study was that of perceived stigmatization. In most of the sessions, participants alluded to being judged in some form by media, strangers and even friends and family. They felt that such opinions were unfair, painful and disrespectful. These findings have been reported in other studies [28,31].

The study has certain limitations. While the diversity of the sample with regard to gender, ethnicity, and education is an important strength of the study, we were unable to conduct separate FGDs for these groups as young people were unwilling to wait for uncertain periods of time before being scheduled for the FGD. We may have uncovered gender or ethnic differences in smoking behavior if we had conducted separate groups. The wide age range (15 to 29 years) of the participants, as well as their length of smoking experience (1 to 16 years), is another limitation of this study. The factors that influence tobacco use in young adolescents may vary considerably with someone in their late 20s. Similarly, the attitudes and issues faced by those who have recently initiated tobacco use are likely to differ considerably from those who had smoked for more than 10 years. Furthermore, including such a diverse age range in the same focus groups may have had an influence on the responses that were given by participants. We were cognizant and had carefully considered these concerns before initiating the FGDs. One of the roles of the note taker was to note if any specific group (based on gender, ethnicity or age-group) was reluctant to express their views or had notably different views. We were open to the idea of conducting focus groups based on specific groups even though this would be time and resource consuming. We debriefed multiple times (both immediately after the FGD and as a group later) to ensure that we were neither missing out the point of view of any group nor inadequately exploring themes there were unique to a group. We do acknowledge that despite our best efforts this remains a limitation of our study. Social desirability may have biased participants’ responses and led them to self-censor their actual views. Lastly, as this is a qualitative study and given the non-representative nature of the sample, these findings cannot be generalized to all young people who smoke. However, it is important to point out that our themes concurred with those of other studies that examined smoking behaviors [28,30].

## 5. Implications for Future Research

Our qualitative study on young people’s smoking behavior identified key themes that are associated with smoking initiation and maintenance in young people. The study has implications for both future research and practice. Family influences on youth smoking are significant and hence parental involvement and education must be a part of prevention programs. Families should be educated with the concept of “anti-smoking socialization”, which has been defined as “the transmission of knowledge, attitudes and skills that prepare children to resist smoking” [35]. This includes establishment and communication of no smoking rules at home, health risks associated with smoking, and the negative consequences of smoking.

The study also suggests that the communication of risk must be in line with young people’s perception of risks and not health risks alone which they tend to minimize and ignore. The Food and Drug Authority, USA [36], launched its first public education campaign to prevent and reduce youth tobacco use, called the “The Real Cost” campaign which focuses on creating awareness of consequences that are relevant to youths such as wrinkling of skin and effects on gums and teeth. Most respondents felt that there was discrimination against smokers and they were stereotyped as unproductive. While this led to some angst, the youth did not mention it as a possible influence for quitting. Prevention strategies should therefore ensure that youth smokers are not further stigmatized. Young smokers lack of self-control and effective stress-management strategies, which emerged as intrapersonal factors, are important from a preventive perspective. Young people must be taught positive ways to cope with stress instead of negative stress management which includes substance use. Positive coping skills can be developed using many strategies such as assertiveness training, learning relaxation techniques and practicing social interaction skills [37]. Schools should continue to focus on developing these life skills in young people.

Lastly, it is possible that youth who stated that smoke-free policies need to be accompanied with smoking cessation programs, may be more likely to consider quitting if such programs are made accessible. Scientifically rigorous interventions, such as motivation enhancement strategies and cognitive behavior therapy, should be made easily available and at affordable prices to support young people in their efforts to quit smoking [38]. The effectiveness of these interventions should also be assessed in the local context.

## 6. Conclusions

In conclusion, this qualitative study on a multi-ethnic sample of young people in an Asian country largely identified themes that are similar to that reported in other qualitative studies conducted in other countries and cultures. Surprisingly, policies did not emerge strongly in the FGDs as a theme given the extremely strict laws that govern the sale of cigarettes and smoking, in Singapore. Despite the robust ‘fear-appeal’ counter-advertising with graphic images of diseased organs as warning labels on every cigarette pack, these young Singaporeans did not acknowledge the harms of smoking more explicitly. The study verified that the TTI model provides a promising theoretical framework to understand smoking initiation and maintenance. It also provides actionable information for initiatives to prevent smoking in young people which includes their perspectives and emphasizes on an inclusive approach without stigmatizing those who smoke.
